# Feeling, caring, knowing: different types of empathy deficit in boys with psychopathic tendencies and autism spectrum disorder

**DOI:** 10.1111/j.1469-7610.2010.02280.x

**Published:** 2010-11

**Authors:** Alice P Jones, Francesca GE Happé, Francesca Gilbert, Stephanie Burnett, Essi Viding

**Affiliations:** 1 Department of Psychology, Goldsmiths CollegeUniversity of London, UK; 2 Division of Psychology and Language SciencesUniversity College London, UK; 3 MRC Social Genetic and Developmental Psychiatry Centre, Institute of PsychiatryKings College London, UK; 4 Institute of Cognitive NeuroscienceUniversity College London, UK

**Keywords:** Psychopathic tendencies, autism spectrum disorder, empathy, cognitive perspective taking

## Abstract

**Background:**

Empathy dysfunction is one of the hallmarks of psychopathy, but it is also sometimes thought to characterise autism spectrum disorders (ASD). Individuals with either condition can appear uncaring towards others. This study set out to compare and contrast directly boys with psychopathic tendencies and boys with ASD on tasks assessing aspects of affective empathy and cognitive perspective taking. The main aim of the study was to assess whether a distinct profile of empathy deficits would emerge for boys with psychopathic tendencies and ASD, and whether empathy deficits would be associated with conduct problems in general, rather than psychopathic tendencies or ASD specifically.

**Methods:**

Four groups of boys aged between 9 and 16 years (*N* = 96) were compared: 1) psychopathic tendencies, 2) ASD, 3) conduct problems and 4) comparison. Tasks were included to probe attribution of emotions to self, empathy for victims of aggression and cognitive perspective-taking ability.

**Results:**

Boys with psychopathic tendencies had a profile consistent with dysfunctional affective empathy. They reported experiencing less fear and less empathy for victims of aggression than comparison boys. Their cognitive perspective-taking abilities were not statistically significantly different from those of comparison boys. In contrast, boys with ASD had difficulties with tasks requiring cognitive perspective taking, but reported emotional experiences and victim empathy that were in line with comparison boys. Boys with conduct problems did not differ from comparison boys, suggesting that the affective empathy deficit seen in boys with psychopathic tendencies was specific to that group, rather than common to all boys with conduct problems.

**Conclusions:**

Although both groups can appear uncaring, our findings suggest that the affective/information processing correlates of psychopathic tendencies and ASD are quite different. Psychopathic tendencies are associated with difficulties in resonating with other people’s distress, whereas ASD is characterised by difficulties in knowing what other people think.

The term ‘empathy’ is used in a variety of ways, and problems of empathy have been suggested to be central to both psychopathy (Hare, 2003) and autism spectrum disorders (ASD; Baron-Cohen & Wheelwright, 2004). However, the ability to resonate with or recognise others’ inner states likely involves a number of potentially separable affective/information processes, and may break down in a number of distinct ways. Important candidate processes include the ability to emotionally ‘resonate’ with other’s feelings while understanding that they are distinct from one’s own (‘affective’ empathy), and the ability to identify what others are thinking or feeling, without necessarily ‘resonating’ with that feeling state (cognitive perspective taking) (de Vignemont & Singer, 2006). The present study compares directly the profile of abilities and difficulties in processes related to affective empathy and cognitive perspective taking in children with psychopathic tendencies (i.e., children who exhibit antisocial behaviour coupled with callous-unemotional (CU) traits) and children with ASD.

Abnormal affective empathy is one of the key hallmarks of psychopathy. The gold standard measurement of psychopathy in adults, the Psychopathy Checklist – Revised (Hare, 2003), refers to ‘callousness and lack of empathy’ and indeed, the ability of psychopaths to inflict serious harm on others is itself an indicator of profound disturbance in the appropriate ‘empathic’ response to the distress of another. In children, the Anti-Social Process Screening Device (Frick & Hare, 2001) makes reference to lack of ‘concern about the feelings of others’.

Blair (2008) has proposed that one of the key processes underpinning functional affective empathy is recognition of others’ distress cues (i.e., fear and sadness). Past studies have consistently indicated that, like adults with psychopathy, children and adolescents with psychopathic tendencies have difficulties in recognising fearful and sad facial and vocal expressions (i.e., others’ distress) (e.g., Blair & Viding, 2008; Dolan & Fullam, 2006; Stevens, Charman, & Blair, 2001). Previous research in children and adolescents also suggests that CU traits are inversely associated with verbal reports of victim concern (another proxy index of affective empathy) in response to short stories depicting aggressive acts (Pardini, Lochman, & Frick, 2003) and that children with high levels of CU traits have difficulties in understanding the reasoning behind story characters’ emotions (Anastassiou-Hadjicharalambous & Warden, 2008).

Dadds et al. (2009) have recently used a parent-rated questionnaire to measure how much children cared about and identified another’s feelings. They found that CU traits were associated with caring less about another’s feelings. In under 9-year-old boys high levels of CU traits were also associated with parent-rated difficulties in identifying another’s feelings, i.e., the ability to know how someone else might feel in a situation that would elicit an emotional response. It is unclear, based on this questionnaire data, whether this association was driven by a broader emotional impairment or a true difficulty in cognitive perspective taking.

In sum, psychopathic tendencies appear to be associated with diminished affective empathy; not recognising or responding to others’ distress. Are they also related to difficulties in identifying what others are thinking, that is, cognitive perspective taking? To date, three studies in adults with psychopathy have shown that their ability to take another’s perspective is intact (Blair et al., 1996; Dolan & Fullam, 2004; Richell et al., 2003). This is perhaps not surprising, as one of the characteristics of psychopathy is the ability to manipulate others (Hare, 2003), which requires good cognitive perspective-taking ability. However, we are not aware of any published research examining cognitive perspective-taking ability (devoid of any emotional content) in children with psychopathic tendencies, and this was one focus for the present study.

The profile of empathy deficits associated with psychopathic tendencies/psychopathy appears different from that seen in individuals with autism spectrum disorders. Hans Asperger placed emphasis on the unpleasant behaviour, ‘Autistic acts of malice’ (p. 77), by the children and adults he described in his account of ‘Autistic Psychopathy’^1^ (Asperger, 1944). However, Frith (1991) in her translation and commentary on Asperger’s work suggests that while the behaviours Asperger described were antisocial in nature, the intent may not have been malicious, but instead aimed at eliciting a clear emotional reaction in other people by individuals who find the social world difficult to interpret. Furthermore, to the extent that affective empathy has been studied in this group, the findings suggest that individuals with ASD find distress in others aversive (Blair, 1999; Sigman, Dissanayake, Corona, & Espinosa, 2003). Although they show lower cognitive perspective-taking scores on a well-validated empathy questionnaire (the Interpersonal Reactivity Index; Davis, 1983), they do not have lower scores on affective empathy (Rogers, Dziobek, Hassenstab, Wolf, & Convit, 2007). Rogers et al. (2007) suggested that individuals with ASD have difficulty in understanding the perspective of others and consequently may react in a seemingly cold and uncaring manner in real-life situations. However, if information is presented in a way that enables individuals with ASD to identify others’ point of view, they appear to show as much concern and compassion as typically developing individuals.

In a study investigating the co-occurrence of psychopathic behaviours and ASD in a selective sample of individuals with a diagnosis of ASD who also displayed antisocial behaviour, it was shown that psychopathic tendencies could co-occur with ASD, a so-called ‘double hit’ (Rogers, Viding, Blair, Frith, & Happé, 2006). The children who had both ASD and psychopathic tendencies showed a profile of being impaired in both affective empathy and cognitive perspective-taking measures, whereas children with ASD without psychopathic tendencies only showed impairments in cognitive perspective taking. Affective empathy deficit was not characteristic of ASD in general, even in this selected sample with high levels of antisocial behaviour. There is also evidence to suggest that psychopathic tendencies and ASD traits, despite both being highly heritable, have a large degree of genetic independence (Jones et al., 2009).

The available data thus suggest that, although both psychopathy and ASD are associated with social difficulties and a decreased outward show of emotions, the aetiology, broad behavioural profiles and the cognitive-affective deficits associated with these two ‘empathy disorders’ may be quite separate (see also Blair, 2008). The primary aim of the current study was to provide the first direct comparison of boys with psychopathic tendencies and boys with ASD on a range of tasks assessing processes related to affective empathy and cognitive perspective taking. We wanted to examine the following questions that are currently outstanding in the field. Firstly, do boys with psychopathic tendencies show a different profile of empathy/cognitive perspective-taking deficits from children with ASD? Specifically, are deficits in abilities related to affective empathy specific to boys with psychopathic tendencies, and deficits in cognitive perspective taking unique to children with ASD? Secondary aims of this study were to examine a) whether boys with psychopathic have difficulty in attributing distress and guilt emotions to self; and b) whether emotion processing and empathy deficits in boys with psychopathic tendencies are unique to this group, or whether they are a merely a by-product of antisocial behaviour and also present in other individuals with conduct problems.

Our first prediction was that boys with psychopathic tendencies would have deficits in the tasks relating to affective empathy, whereas boys with ASD would not show the same profile of difficulties. Secondly, given that children with psychopathic tendencies and psychopathic adults have difficulties attributing distress emotions to others, we made a novel, exploratory prediction that they may also have difficulties attributing these emotions to themselves. Furthermore, we predicted that these deficits would be specific to the emotions of distress; fear and guilt and not encompass other simple and self-conscious emotions (i.e., happiness, disgust or embarrassment). Thirdly, we predicted that cognitive perspective-taking deficits would be specific to the group with ASD. Finally, we predicted that boys with conduct problems (but without psychopathic tendencies) would not show difficulties in affective empathy or attributing emotions to self.

## Method

**Recruitment.** This research was approved by the Institute of Psychiatry and Maudsley Research Ethics Committee. All children were recruited via opportunity sampling using existing school contacts. Active parental consent procedures with child verbal assent were followed for all participants from the mainstream and ASD settings. A ‘safe-guarded’ passive parental consent procedure was exceptionally approved for Emotional and Behavioural Difficulties (EBD) schools in this study. In the EBD setting a passive consent from parents was supplemented with consent given ‘in loco parentis’ by class teachers. In all cases we also obtained verbal assent from the boys taking part in the study. This procedure was approved by the Ethics Committee on the basis that students attending EBD schools often have chaotic home lives with documented failure to communicate with the schools. Many boys recruited from EBD settings boarded at school or were under the care of local authorities. Recruiting only those individuals with active written parental consent would have therefore resulted in an extremely selective and unrepresentative sample.

In total, five parents or carers of pupils attending EBD schools indicated that they would prefer their child not to take part in the study and these boys were not approached for testing. Ninety-one percent of the EBD boys who were approached took part in research. All the boys in the ASD settings received active parental consent to take part in the research. Four mainstream classes (with 54 boys in total) that corresponded to the age range of the EBD and ASD boys were approached last. Seventy-four percent of boys received active parental consent to take part in the research, but four boys were not tested owing to being absent from school on the day of testing.

**Participants:** This study focused on boys as both conduct problems and ASD are more common in males. The 110 participants were aged between 9y 3m and 16y 9m and were from three mainstream schools in low socioeconomic status (SES) areas (in London, Essex, and Stockport; *N* = 36), from five schools for boys with emotional and behavioural difficulties (in Hertfordshire, Surrey, Oxford and Stockport; *N* = 51), and from four special units for students with ASD (in London and Buckinghamshire; *N* = 23). Eight boys were excluded owing to low full-scale IQ (FSIQ) (<70; 2 with ASD). The remaining 102 boys were split into four groups.

## Group selection criteria

Selection criteria for each group were as follows:

1. Psychopathic tendencies: Scores of 50 or above on Conduct Disorder scale of the CSI or ASI^2^ and a score of 32 or above on the ICU^3^.
2. Conduct problems: Scores of 50 or above on Conduct Disorder scale of the CSI or ASI and a score of 31 or below on the ICU.
3. ASD: Participants with ASD were recruited from schools with specialist provision for students with ASD. All participants had received a diagnosis of autism, Asperger’s Disorder or atypical autism from a psychiatrist or paediatrician and had a Statement of Special Educational Needs stating this as the primary disorder.^4^
4. Comparison group: Boys in the comparison group were required to score under 50 on the CSI or ASI and below 32 on ICU.

Six boys who did not fulfil the criteria for any of the groups (elevated ICU scores, above 31 and low CP scores, below 50) were excluded at the group selection stage. The final sample size for this study was 96: 21 boys with psychopathic tendencies, 21 with ASD, 23 with conduct problems and 31 comparison boys.

## Measures

**Inventory of Callous/Unemotional Traits (ICU; Frick, 2003).** The ICU is a 24-item scale, which has been previously shown to delineate a distinct and important group of antisocial youths who show a number of characteristics associated with the construct of psychopathy. This measure has been shown to have acceptable internal consistency (alpha = .87 in this study; in others: alpha = .77; Essau, Sasagawa, & Frick, 2006; alpha = .81; Viding, Simmonds, Petrides, & Frederickson, 2009). This instrument also shows good construct validity in school (Essau et al., 2006; Viding et al., 2009) and adolescent offender (Kimonis et al., 2008) samples. We used a teacher rather than parent-report version of the ICU in this study because some of the participants came from chaotic home backgrounds, where parental non-response was considered to be likely.

**Child Symptom Inventory IV and Adolescent Symptom Inventory IV (ASI; Gadow & Sprafkin, 1997; CSI; Gadow & Sprafkin, 2002).** These measures have been designed to screen for the behavioural, emotional and cognitive symptoms of DSM-IV disorders (American Psychiatric Association, 1994). The Child’s Symptom Inventory (CSI) is for use with children aged 5–12 years old, and the Adolescent Symptom Inventory (ASI) is for individuals aged 12–18 years. This study used the Conduct Disorder scale from the CSI and ASI. All Conduct Disorder behaviours assessed using CSI or ASI were rated by the teachers as occurring ‘never’, ‘sometimes’, ‘often’ or ‘very often’. The items for the CSI and ASI Conduct Disorder scales are the same, but the translation of raw scores to t-scores uses different age norms. It is the t-score from this scale that is used in the grouping criteria.

**Ability.** To give an estimate of general cognitive ability, the short-form of the Wechsler Abbreviated Scales of Intelligence (WASI; Wechsler, 1999) was used. This includes assessment of Vocabulary and Matrix Reasoning. Full-scale IQs (FSIQ) are reported in Table 1.

**Table 1:** Participants’ characteristics by group

	PT		CP		C		ASD				
	Mean	(SD)	Mean	(SD)	Mean	(SD)	Mean	(SD)	*F*	*p*	Post-hoc
*N*	21		23		31		21				
Age (yrs;ms)	12;4	(1;10)	11;9	(1;2)	11;6	(1;5)	13;1	(1;8)	4.74	.004	ASD > C
Age range	9y10m–16y 9m		9y 5m–15y 10m		9y 3m–16y 3m		10y 7m–16y 5m		
Conduct Problems (CP) *t*-score	77.57	(20.66)	60.87	(7.49)	46.13	(.92)	N/A	N/A	25.07	<.001	PT > CP > C
Psychopathic tendencies (PT)	41.74	(6.45)	25.88	(4.57)	20.00	(5.43)	26.81	(9.66)	47.83	<.001	PT > CP/ASD > C
Full Scale IQ (FSIQ)	87.00	(12.73)	91.26	(11.51)	99.81	(13.08)	94.33	(11.59)	4.92	.003	C > PT

PT = Psychopathic tendencies, CP = conduct problems, C = comparison, ASD = autism spectrum disorder.

**Outcome Values Measure (Boldizar, Perry, & Perry, 1989; Pardini et al., 2003).** This measure consists of eight vignettes designed to assess the values that participants place on the outcomes of reactive and proactive aggression against a same-sex peer. For the reactive aggression items, participants were asked to rate how much they cared about 1) reducing the aversive behaviour of the peer; 2) being punished for their aggressive response; 3) making the peer feel bad; 4) feeling bad for their own actions; and 5) gaining a sense of dominance from their actions. For the vignettes depicting instrumental aggression participants were also asked how much they would care about obtaining the desired item. Outcomes 2–5 were the same as for the reactive aggression items. After each vignette, participants were asked to rate how much they would care if five specific outcomes occurred as a result of their behaviour. Ratings were on a four-point scale (1 = ‘I would not care at all’ to 4 = ‘I would care a lot’). Total scores were entered into subsequent analyses (maximum possible score = 16). This instrument has been shown to discriminate reliably between aggressive and non-aggressive youths, with aggressive youth reporting that they would care less about their victim’s feelings (Hall, Herzberger, & Skrowronski, 1998), and has also shown an association with psychopathic tendencies in a sample of juvenile delinquents, where psychopathic tendencies were associated with lower values placed on the negative outcomes of aggressive acts (Pardini et al., 2003).

**Emotion Attribution to Self (Burnett, Bird, Moll, Frith, & Blakemore, 2009).** This task was developed to assess attributions of emotions to self. The task was presented as a modified version of the questionnaire used by Burnett et al. (2009), containing eight situations assessing four different emotions (fear, disgust, embarrassment and guilt). The task was originally piloted on over 80 children and only those vignettes which were clearly rated as depicting a specific emotion were selected. Four happy filler items were also included in the questionnaire to ensure that participants did not solely think about negative content during the task. Participants were given a scenario and asked to rate how much of a corresponding emotion they would feel in that situation. For example, ‘You made fun of a quiet girl you know and it made her cry. How *guilty* would you feel?’ Participants could select whether they would ‘not feel this emotion at all’, ‘feel this emotion a bit’, ‘feel this emotion quite a bit’ or ‘feel this emotion a lot’. These responses were scored 1–4 respectively.

**First- and Second-Order Theory of Mind (Baron-Cohen, 1989; Bowler, 1992).** This task was included to assess first- and second-order theory of mind (ToM), and was based on tasks by Baron-Cohen (1989) and Bowler (1992). This task has been shown to have good test–retest reliability (Hughes et al., 2000) and concurrent validity (Sullivan, Zaitchik, & Tager-Flusberg, 1994). The first-order false belief question required reasoning about what another person might mistakenly think. The second-order false belief question required reasoning about what one person mistakenly thinks another person thinks. Participants were told a short story using pictures about a boy and a girl who had been given some chocolate to share. When the girl was out of sight, the boy hid the chocolate in his bag. Participants were asked where the girl thought the chocolate was at that point (first-order ToM). It was then revealed that the girl had in fact seen (through the window) the boy take the chocolate out of the fridge and put it in his bag, although the boy did not know. The participant was asked where the boy thought the girl would look for the chocolate (second-order ToM). Participants were asked to justify their response, as well as being asked two control questions about where the chocolate was really, and where it was initially. Participants could score a maximum of 14 points, with two points being awarded for a fully correct answer, one point for a partially correct answer, and no points for a wrong response to the justification questions.

**Theory of Mind Animation Task (Abell, Happé, Frith, & Frith, 2000; Castelli, Frith, Happé, & Frith, 2002).** This task has shown differences in ToM abilities between child samples with and without ASD (Abell et al., 2000; Campbell et al., 2006) and has been demonstrated to show convergent validity with other tasks where there is typically a deficit in individuals with ASD (Campbell et al., 2006). Participants were shown four animations on a laptop computer, all featuring two animated triangles: one large red triangle and one small blue triangle. Interaction between the two triangles was scripted to imply complex mental states such as the intention to ‘trick’ another character. These animations could thus be described in terms of the characters’‘thoughts’. Participants were asked to describe what had happened after each animation. These commentaries were scored on two scales, ‘Intentionality’ and ‘Appropriateness’, according to an established, graded scoring system. Intentionality was scored on a scale of 0–5 (where 0 = non-deliberate action, and 5 = deliberate action with the intention of affecting another’s mental state). Appropriateness is scored on a scale of 0–2 (according to the perceived understanding of the animation as intended by the authors). The scoring criteria are the same as those given in Castelli et al. (2002)

## Procedure

All participants were tested in a quiet room on the school premises. Testing sessions were negotiated with the teacher so as to minimise interference to the participant’s learning and to the class. All participants were read an information sheet and were given an opportunity to ask questions. They were also informed that it was ok to stop their session at any time. All participants gave verbal assent to take part in this study. All tasks except for the WASI and first- and second-order ToM task were administered on a laptop computer. All participants were tested using the WASI first, followed by the other tasks in a random order.

## Data analysis

The four groups were entered into task-specific ANOVAs. As recommended in the literature (e.g., Miller & Chapman, 2001; Knight & Silverstein, 2001), we do not report covariate analyses, as such analyses are considered problematic to interpret. However, where age or FSIQ were related to variables of interest, the group difference reported below remained even after co-varying for these variables (see online appendix). In short, group differences cannot be attributed to group differences in age or IQ. All statistically significant group differences were followed up by post-hoc Tukey HSD tests. Corrections for multiple comparisons (Tukey HSD) are made for the number of between-group analyses.

## Results

### Participant characteristics

Participant characteristics are presented in Table 1. As expected, the psychopathic tendencies group had significantly greater ICU scores than all other groups. They also had the greatest level of conduct problems, having statistically significantly greater scores than all other groups. The CP group had statistically significantly greater conduct problem scores than the group with ASD and the comparison group. Boys with ASD were statistically significantly older than the comparison group only; no other group differences for age were statistically significant. Finally, although comparison boys were recruited from similar educational backgrounds to the boys with high levels of conduct problems, there was a statistically significant group difference in FSIQ between comparison boys and boys with psychopathic tendencies; no other group differences for FSIQ were statistically significant.

### Outcome values

**Figure 1:**
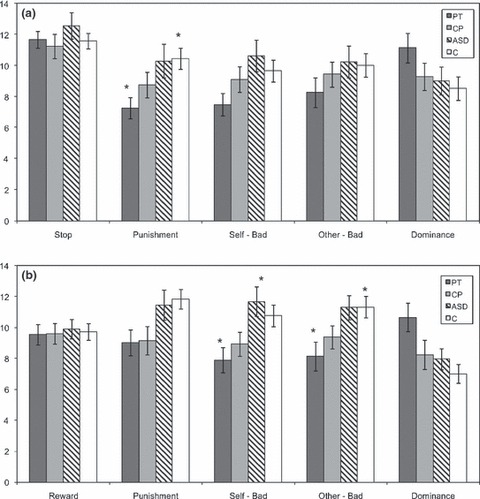
Bar chart of total scores for outcome values: a) vignettes of reactive aggression, b) vignettes of instrumental aggression (* indicates significant differences between groups, *p* < .05 that remain after co-varying for age and IQ). PT = psychopathic tendencies, CP = conduct problems, C = comparison, ASD = autism spectrum disorder

The outcome values scores are presented in Figure 1. For the reactive aggression vignettes, there was a statistically significant main effect of group for the ‘caring about being punished for the reactively aggressive action’ (*F*_(3, 84)_ = 3.10, *p* = .03). Tukey HSD tests revealed that the group with psychopathic tendencies cared significantly less than comparison boys about being punished for their actions (*p* = .04).

The outcome values attributed to incidences of instrumental aggressive acts also differed significantly between the groups. There were significant group differences for the items about making the victim feel bad (*F*_(3, 84)_ = 3.77, *p* = .01), where boys with psychopathic tendencies reported that they would care significantly less about the victim’s feelings than comparison boys (*p* = .02). There was also a statistically significant group difference for the social dominance items (*F*_(3, 84)_ = 3.62, *p* = .02). Boys with psychopathic tendencies reported that they would place greater value on being ‘boss’ of the situation than comparison participants (*p* = .01). Finally, there was a statistically significant group difference regarding feeling bad about own behaviour (*F*_(3, 84)_ = 4.08, *p* = .01). Post-hoc Tukey HSD tests indicated that the group with psychopathic tendencies reported that they would feel significantly less bad about their behaviour compared with boys with ASD (*p* = .02).

### Emotion attribution to self

Results from this task are presented in Table 2. A statistically significant group difference was found for the self-attribution of fear (F^(3, 81)^ = 4.66, *p* < .01). In line with predictions, post-hoc Tukey HSD tests indicated that the group with psychopathic tendencies self-attributed fear statistically significantly less than the comparison group (*p* < .01). There were no statistically significant group differences for self-attributions of guilt, disgust, embarrassment or happiness, although there was a trend for guilt, with the group with psychopathic tendencies showing the lowest levels of self-attribution of guilt.

**Table 2:** Self-attribution of emotion scores by group (maximum score = 4)

	PT		CP		C		ASD				
	Mean	(SD)	Mean	(SD)	Mean	(SD)	Mean	(SD)	*F*	*p*	Post-hoc
Fear	2.28	(.89)	2.54	(.87)	2.90	(.70)	3.09	(.70)	4.29	<.01	C, ASD > PT
Guilt	2.89	(.81)	3.27	(.84)	3.35	(.59)	3.43	(.57)	2.35	.08	–
Happiness	3.14	(.90)	3.38	(.73)	3.42	(.50)	3.50	(.60)	1.04	.38	–
Disgust	3.41	(.70)	3.52	(.72)	3.42	(.47)	3.60	(.54)	.47	.70	–
Embarrassment	3.04	(.90)	3.10	(.93)	3.13	(.70)	3.03	(.73)	.06	.98	–

PT = psychopathic tendencies, CP = conduct problems, C = comparison, ASD = autism spectrum disorder.

### Theory of mind

There were statistically significant group differences for both the first- and second-order ToM tasks and the Intentionality subscale of the Animations task (*F*_(3, 85)_ = 3.49, *p* = .02 and *F*_(3, 77)_ = 3.59, *p* = .02 respectively) . On the first- and second-order ToM task the group with psychopathic tendencies scored at ceiling. There were no statistically significant differences between the group with psychopathic tendencies and comparison boys. However, post-hoc Tukey HSD tests indicated that the group with psychopathic tendencies scored significantly higher than the ASD group (*p* = .03), as did the comparison group (*p* = .05). For the ToM animations task, there were no statistically significant differences between the group with psychopathic tendencies and the comparison group. However, post-hoc Tukey HSD tests indicated that the comparison group scored significantly higher than the ASD group (*p* = .01). Scores for the ToM tasks are presented in Table 3.

**Table 3:** Theory of mind scores by group

	PT		CP		C		ASD				
	Mean	(SD)	Mean	(SD)	Mean	(SD)	Mean	(SD)	*F*	*p*	Post-hoc
Theory of mind											
1st & 2nd order	14.00	(.00)	13.65	(.99)	13.93	(.27)	13.2	(1.51)	3.49	.02	C, PT > ASD
Animations: Intentionality	12.26	(2.68)	13.16	(2.57)	13.75	(2.19)	11.19	(2.90)	3.59	.02	C > ASD
Animations: Appropriateness	3.95	(1.65)	4.26	(1.48)	4.42	(1.10)	3.93	(1.44)	.57	.63	-

PT = psychopathic tendencies, CP = conduct problems, C = comparison, ASD = autism spectrum disorder.

## Discussion

This was the first study to demonstrate that the types of ‘empathy deficit’ characteristic of psychopathic tendencies and ASD are specific to each psychopathology, and that these ‘empathy deficits’ are not shared with boys who have conduct problems without callous-unemotional traits.

Boys with psychopathic tendencies had specific deficits in domains associated with affective empathy. The outcome values task enabled us to investigate how much boys with psychopathic tendencies care about the consequences of aggressive actions on a victim. This task might be thought of as a proxy measure of affective empathy (as defined by Dadds et al., 2009) asking specifically how much the participant would care about the outcomes of the aggressive act, including the feelings of the victim. Although this measure provides us with only an insight into this particular form of affective empathy deficit (that is, caring whether they have hurt somebody else), it is notable that this deficit in boys with psychopathic tendencies was particularly apparent for the deliberate instrumental aggression scenarios. Here, they reported caring less than comparison boys and boys with ASD about their own feelings, as well as the feelings of the scenario victims. We also showed that boys with psychopathic tendencies attributed significantly less fear to themselves than comparison boys. This finding is in line with studies showing decreased autonomic nervous system responses to fearful stimuli and difficulties recognising distress emotions in others for children with psychopathic tendencies and adults with psychopathy (Aniskiewicz, 1979; Blair, Jones, Clark, & Smith, 1997; Blair, Colledge, Murray, & Mitchell, 2001; Dolan & Fullam, 2006; Stevens et al., 2001). There were no statistically significant group differences on any of the other simple or self-conscious emotions tested by this task, although there was a trend for guilt. A previous study on adult psychopaths reported that this group has difficulty attributing guilt to a story protagonist (Blair et al., 1995). We speculate that the current task may not be sensitive enough to detect the ‘lack of guilt’ that is associated with psychopathic tendencies. This could be due to the fact that the task asked about guilt explicitly and there may have been an attempt on the part of some youth to present socially desirable and acceptable responses to an outright query about potential feelings of guilt. The previous study of emotion attribution in adult psychopaths reports low guilt attribution to others (Blair et al., 1995), a condition under which the social desirability may be less evident than in our study. Lack of guilt attribution to self is in contrast with the outcome values task where questions probing guilt and empathy were less direct and focused on caring about a specific outcome.

In line with predictions, deficits in cognitive perspective-taking ability, as measured by two ToM tasks, were found to be specific to the group with ASD. Boys with psychopathic tendencies were found to have cognitive perspective-taking skills that were not statistically significantly different from those of the typically developing comparison group. These findings are in line with data on adult psychopaths suggesting intact cognitive perspective-taking skills (Dolan & Fullam, 2004; Richell et al., 2003). In contrast, the group with ASD showed deficits on both cognitive perspective-taking tasks employed in this study. However, it is important to note that in one of our tasks (the first- and second-order ToM task), all groups scored very close to ceiling and future research would benefit from using additional tasks, besides the triangles task, that are not prone to such ceiling effects. It is of interest to note that for the outcome values and emotion attribution tasks, there was no need to work out what the other person was thinking. This information was always given in an explicit way such as ‘you forgot your friend’s birthday and made him feel sad’. This means that any difficulties with cognitive perspective taking for the group with ASD were circumvented, and as such these tasks afforded a snapshot of the ASD individuals’ capacity for affective empathy independent of difficulties with cognitive perspective taking.

Although these findings offer promising new research leads, it is critical to acknowledge the limitations of this study. The *N* for each group was modest and although our data yield statistically significant results after correcting for multiple comparisons, it is possible that we may have missed relevant effects of smaller magnitude. This sample was also purely community based. Since a formal diagnostic work-up was beyond the scope of this study, we cannot exclude the possibility that some participants attending EBD special school may also meet criteria for an ASD. However, any child attending EBD provision who was already known to have an ASD diagnosis was not recruited for this study and it should be noted that the possible inclusion of such participants would have likely weakened the group difference findings in this study. We were also not able to independently verify the ASD diagnoses, although the children attended special provision for children with ASD and had been diagnosed by clinicians. Future work could extend this research to clinic samples of children with conduct disorder. It may be predicted that individuals who have been referred for clinical interventions might have a more severe profile of difficulties in the area of empathy and emotion attribution. Additional limitation concerns lack of data on other domains of psychopathology, e.g., anxiety, an area where we would expect boys with psychopathic tendencies to show few problems. We therefore cannot rule out a contribution of some other psychopathology to our findings. We also cannot rule out that some of the differences between the two conduct problem groups may be driven by differences in the severity of conduct problems (higher in children with psychopathic tendencies) rather than CU. We emphasise that the boys with conduct problems (without CU) do not differ from controls on any of the measures presented in this study, despite differing significantly with respect to their levels of conduct problem symptoms. However, we accept that this cannot rule out the possibility of a difference emerging only at high levels of conduct problem severity.

There are several interesting possibilities for future research in this area. The cognitive perspective taking for emotional and non-emotional content could be contrasted in these groups. Developmental trajectories of affective empathy and cognitive perspective taking and how these vary between groups is also of interest and would add to Dadds and colleagues’ work using questionnaire measures (Dadds et al., 2009). The current study offered only a snapshot of these processing domains at a single time-point and each group was comprised of boys of varying ages. There is no doubt room for developing more precise and ecologically valid measures, as well as extending research in this area to direct comparison of biomarkers of empathy in psychopathic tendencies and ASD.

The findings from this line of research have scope to inform the behavioural interventions and clinical practice more generally. Boys with psychopathic tendencies have cognitive perspective-taking skills in line with their typically developing peers, but do not care about the feelings of others in the same way that other boys with conduct problems might. Interventions that use empathy induction techniques may be inappropriate for boys with psychopathic tendencies if these boys do not have the basic capacity to feel for others. Furthermore, training those with psychopathic tendencies in emotion recognition skills may serve only to make these individuals better manipulators of their victims, especially as they appear to have adequate mentalising skills. More work is necessary to build a comprehensive profile of affective/information processing biases associated with psychopathic tendencies and to develop interventions that draw on strengths and avoid weaknesses associated with this condition. It is also of immediate significance to communicate effectively to professionals in clinical practice that although some behavioural features of psychopathic tendencies and ASD overlap, the core deficits associated with each condition appear markedly different.

**Key points**

- Boys with psychopathic tendencies have significant impairments in affective empathy, for example they appear not to care when someone has been hurt. However, boys with psychopathic tendencies do not appear to differ from a comparison group on their cognitive perspective-taking ability – they are good at knowing what someone else is thinking.
- Boys with autism spectrum disorders (ASD) have significant impairments in their cognitive perspective-taking ability; in other words, they have difficulty knowing what someone else is thinking. Although they often react in a socially inappropriate manner, boys with ASD appear to feel the expected affective response to other people’s distress and to have intact affective empathy.
- Boys with conduct problems without psychopathic tendencies do not differ from a comparison group on their capacity to care about others’ feelings or their cognitive perspective-taking ability.

## Footnotes

1 Psychopathy in this instance refers to psychopathology rather than the current clinical conceptualisation of psychopathy.

2 A cut-off of a *t*-score of 50 or above was selected to ensure that all participants with conduct problems or psychopathic tendencies were rated as having higher than average levels of conduct problems. All comparison participants were selected to score under 50.

3 The groups with psychopathic tendencies and conduct problems were split according to the median ICU score for children with CP.

4 Although ICU and CSI/ASI were not used to recruit the ASD group, ICU ratings had been collected by the school for this group and enabled comparison on these traits. Six participants with ASD scored at or above the cut-off of 33. Removing these individuals from the analyses did not change the pattern of results, and since their inclusion would potentially *reduce* the predicted group differences, they are retained in the sample analysed below.

## Abbreviations

ASD: autism spectrum disorders

## Supplementary material

The following supplementary material is available for this article:

Appendix 1 Correlations and ANCOVAs with age and IQ for tasks with significant group differences (Word document) This material is available as part of the online article from: http://www.blackwell-synergy.com/doi/abs/10.1111/j.1469-7610.2010.02280.x Please note: Blackwell Publishing are not responsible for the content or functionality of any supplementary materials supplied by the authors. Any queries (other than missing material) should be directed to the corresponding author for the article.

## References

[b1] Abell F, Happé F, Frith U, Frith C (2000). Do triangles play tricks? Attribution of mental states to animated shapes in normal and abnormal development. Journal of Cognitive Development.

[b2] American Psychiatric Association (1994). Diagnostic and statistical manual of mental disorders.

[b3] Anastassiou-Hadjicharalambous X, Warden D (2008). Cognitive and affective perspective-taking in conduct-disordered children high and low on callous-unemotional traits. Child and Adolescent Psychiatry and Mental Health.

[b4] Aniskiewicz A (1979). Autonomic components of vicarious conditioning and psychopathy. Journal of Clinical Psychology.

[b5] Asperger H, Frith U (1944). Die ‘Autistischen Psychopathen’ im Kindesalter [Autistic Psychopathy of Childhood, trans. U. Frith (1991)]. Autism and Asperger syndrome.

[b6] Baron-Cohen S, Wheelwright C (2004). The Empathy Quotient (EQ). An investigation of adults with Asperger Syndrome or High Functioning Autism, and normal sex differences. Journal of Autism and Developmental Disorders.

[b7] Blair R (1999). Psychophysiological responsiveness to the distress of others in children with autism. Personality & Individual Differences.

[b8] Blair RJ (2008). Fine cuts of empathy and the amygdala: Dissociable deficits in psychopathy and autism. Quarterly Journal of Experimental Psychology.

[b9] Blair R, Colledge E, Murray L, Mitchell D (2001). A selective impairment in the processing of sad and fearful expressions in children with psychopathic tendencies. Journal of Abnormal Child Psychology.

[b10] Blair RJ, Jones L, Clark F, Smith M (1997). The psychopathic individual: A lack of responsiveness to distress cues?. Psychophysiology.

[b11] Blair RJR, Sellars C, Strickland I, Clark F, Williams AO, Smith M (1995). Emotion attributions in the psychopath. Personality and Individual Differences.

[b12] Blair RJR, Sellars C, Strickland I, Clark F, Williams A, Smith M (1996). Theory of mind in the psychopath. Journal of Forensic Psychiatry.

[b13] Blair RJR, Viding E, Rutter M, Bishop D, Pine D, Scott S, Stevenson J, Taylor E, Thapar A (2008). Psychopathy. Rutter’s child and adolescent psychiatry.

[b14] Boldizar JP, Perry DG, Perry LC (1989). Outcome values and aggression. Child Development.

[b15] Bowler DM (1992). ‘Theory of mind’ in Asperger’s syndrome. Journal of Child Psychology and Psychiatry.

[b16] Burnett S, Bird G, Moll J, Frith C, Blakemore SJ (2009). Development during adolescence of the neural processing of social emotion. Journal of Cognitive Neuroscience.

[b17] Campbell R, Lawrence K, Mandy W, Mitra C, Jeyakuma L, Skuse D (2006). Meanings in motion and faces: Developmental associations between the processing of intention from geometrical animations and gaze detection accuracy. Development and Psychopathology.

[b18] Castelli F, Frith C, Happé F, Frith U (2002). Autism, Asperger syndrome and brain mechanisms for the attribution of mental states to animated shapes. Brain.

[b19] Dadds MR, Hawes DJ, Frost AD, Vassallo S, Bunn P, Hunter K (2009). Learning to ‘talk the talk: The relationship of psychopathic traits to deficits in empathy across childhood. Journal of Child Psychology and Psychiatry.

[b20] Davis MH (1983). Measuring individual differences in empathy: Evidence for a multidimensional approach. Journal of Personality and Social Psychology.

[b21] De Vignemont F, Singer T (2006). The empathic brain: How, when and why?. Trends in Cognitive Sciences.

[b22] Dolan M, Fullam R (2004). Theory of mind and mentalizing ability in antisocial personality disorders with and without psychopathy. Psychological Medicine.

[b23] Dolan M, Fullam R (2006). Face affect recognition deficits in personality-disordered offenders: Association with psychopathy. Psychological Medicine.

[b24] Essau CA, Sasagawa S, Frick PJ (2006). Callous-unemotional traits in a community sample of adolescents. Assessment.

[b25] Frick PJ (2003). The Inventory of Callous/Unemotional traits: An unpublished rating scale.

[b26] Frick PJ, Hare RD (2001). Antisocial Process Screening Device.

[b27] Frith U (1991). Autism and Asperger syndrome.

[b28] Gadow KD, Sprafkin J (1997). Adolescent Symptom Inventory IV.

[b29] Gadow KD, Sprafkin J (2002). Child Symptom Inventory IV.

[b30] Hall JA, Herzberger SD, Skrowronski KJ (1998). Outcome expectancies and outcome values as predictors of children’s aggression. Aggressive Behavior.

[b31] Hare RD (2003). The Hare Psychopathy Checklist – Revised.

[b32] Hughes C, Adalm A, Happé F, Jackson J, Taylor A, Caspi A (2000). Good test–retest reliability for standard and advanced false-belief tasks across a wide range of abilities. Journal of Child Psychology and Psychiatry.

[b33] Jones AP, Larsson H, Ronald A, Rjisdijk F, Busfield P, McMillan A (2009). Phenotypic and aetiological relationships between psychopathic tendencies, autistic traits, and emotion attribution. Criminal Justice and Behavior.

[b34] Kimonis ER, Frick PJ, Skeem JL, Marsee MA, Cruise K, Munoz LC (2008). Assessing callous-unemotional traits in adolescent offenders: Validation of the Inventory of Callous-Unemotional Traits. International Journal of Law and Psychiatry.

[b35] Knight RA, Silverstein SM (2001). A process-oriented approach for averting confounds resulting from general performance deficiencies in schizophrenia. Journal of Abnormal Psychology.

[b36] Miller GA, Chapman JP (2001). Misunderstanding analysis of covariance. Journal of Abnormal Psychology.

[b37] Pardini DA, Lochman JE, Frick PJ (2003). Callous/unemotional traits and social-cognitive processes in adjudicated youths. Journal of the American Academy of Child & Adolescent Psychiatry.

[b38] Richell R, Mitchell D, Newman C, Leonard A, Baron-Cohen S, Blair R (2003). Theory of mind and psychopathy: Can psychopathic individuals read the ‘language of the eyes’?. Neuropsychologia.

[b39] Rogers J, Viding E, Blair RJ, Frith U, Happé F (2006). Autism spectrum disorder and psychopathy: Shared cognitive underpinnings or double hit?. Psychological Medicine.

[b40] Rogers K, Dziobek I, Hassenstab J, Wolf OT, Convit A (2007). Who cares? Revisiting empathy in Asperger syndrome. Journal of Autism and Developmental Disorders.

[b41] Sigman M, Dissanayake C, Corona R, Espinosa M (2003). Social and cardiac responses of young children with autism. Autism.

[b42] Stevens D, Charman T, Blair R (2001). Recognition of emotion in facial expressions and vocal tones in children with psychopathic tendencies. Journal of Genetic Psychology.

[b43] Sullivan K, Zaitchik D, Tager-Flusberg H (1994). Preschoolers can attribute second-order beliefs. Developmental Psychology.

[b44] Viding E, Simmonds E, Petrides KV, Frederickson N (2009). The contribution of callous-unemotional traits and conduct problems to bullying in early adolescence. Journal of Child Psychology and Psychiatry.

[b45] Wechsler D (1999). Wechsler Abbreviated Scales of Intelligence.

