# Supplementary material for: Feeling, caring, knowing: different types of empathy deficit in boys with psychopathic tendencies and autism spectrum disorder
Source: J Child Psychol Psychiatry. 2010 Nov;51(11):1188–97. doi: 10.1111/j.1469-7610.2010.02280.x (PMC3494975; doi:10.1111/j.1469-7610.2010.02280.x)
Supplement: Appendix 1 — Correlations and ANCOVAs with age and IQ for tasks with significant group differences (Word document) This material is available as part of the online article from: http://www.blackwell-synergy.com/doi/abs/10.1111/j.1469-7610.2010.02280.x Please note: Blackwell Publishing are not responsible for the content or functionality of any supplementary materials supplied by the authors. Any queries (other than missing material) should be directed to the corresponding author for the article. [file jcpp0051-1188-SD1.docx]

[app]**Appendix 1** Correlations and ANCOVAs with age and IQ for tasks with significant group differences

| Task | Correlation with age | ANCOVA (age) | Correlation with IQ | ANCOVA (IQ) |
| --- | --- | --- | --- | --- |
| VS values | | | | |
| Reactive: punishment | *r* = –.25; *p* = .02 | *F* = 3.24; *p* = .03 | *r* = .12; *p* = .27 | n/a |
| Instrumental: self feeling bad | *r* = –.18; *p* = .09 | n/a | *r* = .07; *p* = .56 | n/a |
| Instrumental: other feeling bad | *r* = –.20; *p* = .07 | n/a | *r* = .11; *p* = .32 | n/a |
| Instrumental: dominance | *r* = –.13; *p* = .23 | n/a | *r* = –.32; *p* = .003 | *F* = 2.13; *p* = .10 |
| Emotion attribution | | | | |
| Fear | *r* = –.17; *p* = .11 | n/a | *r* = .25; *p* = .04 | *F* = 3.22; *p* = .03 |
| ToM | | | | |
| First and second order | *r* = –.14; *p* = .20 | n/a | *r* = .16; *p* = .14 | n/a |
| ToM: intentionality | *r* = .08; *p* = .50 | n/a | *r* = .30; *p* = .01 | *F* = 3.37; *p* = .03 |
